# Annual Meeting of the Lake Erie Dental Association

**Published:** 1871-11

**Authors:** 


### Proceedings of Societies.

---

### ANNUAL MEETING OF THE LAKE ERIE DENTAL ASSOCIATION.

The last regular annual meeting of the Lake Erie Dental Association, was held in Meadville, Pa., commencing on the 6th of June, 1871, and continuing for three days. Dr. G. B. McDonneld, President, in the chair.

Minutes of last meeting read, corrected and approved.

The following members were present at the meeting:

President, G. B. McDonneld, of Conneautville; Vice-President, J. G. Templeton, New Castle; Secretary, C. H. Bagley, Meadville,; Treasurer, S. W. Tenney, Corry; Censors, A. B. Robbins, Meadville; J. G. Templeton, New Castle, and C C. Carroll, *pro tem.*, Meadville.

Present—J. A. Marsh, Union Mills; E. R. Allen, Girard; D. C. Dunn, Meadville; C. D. Elliott, Franklin; W. T. Wallace, Kingsville, Ohio; S. W. Tenney, Corry; W. H. H. Brown, Conneautville; J. B. Humes, Edinboro; E. M. Pierce, Warren; F. Herrick, Greenville; Geo. Elliott, Meadville; G. W. Nelson, Ashtabula, O.; J. H. Nelson, North-East; C. H. Griffin, Oil City; C. B Ansart, Oil City; Jas. Harrison, Jamestown, N. Y.; W. M. Martin, Mercer.

Dr. Robbins delivered an address of welcome, in which he gave a brief history of the dental profession in Meadville, claiming for a citizen of this city the original use of the mallet when filling teeth, viz.: Dr. Beatty, who drove in his filling with a shoe hammer; showing the great progress and improvement of dentistry since the days when Drs. Merritt and Wright sojourned here on their periodical trips from Pittsburg to Erie, and when a German used to travel through this place advertising his "Royal Mineral Succedaneum, a composition superior to gold for filling teeth," which proved to be a simple amalgam; and finally illustrated from his personal experience the great advance of the profession in liberality. Formerly all knowledge was kept secret as far as possible; now all freely receive and freely give.

The President next addressed the meeting. The central thought of the address, dwelt upon at some length, was the mighty force of associated effort in the search after knowledge. The writer called attention to the failure of the committee appointed to obtain a State law regulating the practice of dentistry, and urged the society to renewed efforts through petitions and other channels, as essential to success.

The following persons were then nominated for membership: Dr. Kolb, of Franklin; Dr. M. B. Naramore, of Linesville; Dr. W. M. Martin, of Mercer; Dr. W. H. Reynolds, of Rouseville, and Dr. H. and Mrs. E. Wormersley, of Meadville.

Dr. Gifford being absent, Dr. Carroll was appointed to fill his place on the Board of Censors.

Dr. Martin, favorably passed upon by the board, was balloted for and duly elected a member of the association.

Dr. Robbins announced the death of Dr. R. G. Snow, of Buffalo, member of the association. Suitable action was taken expressing regret for his loss.

The Constitution and By-laws revised by special committee, were reported and adopted.

### REPORTS OF DELEGATES.

The chair announced that reports of delegates were in order.

Dr. Robbins, though not a delegate, reported that the meeting of the State Society last year in Pittsburg, brought about by this Society, had aroused the profession in that city to renewed efforts and improved practice, and had infused new life into the local society there.

### DENTAL CHEMISTRY.

Dr. Elliott being called on, read an essay on "Dental Chemistry."

Remarks and discussions followed.

Dr. Wormersley, one of the visitors, doubts that fluids of the mouth affect the teeth, but thinks that they are destroyed by temperature and climate.

Dr. C. D. Elliott thought although the fluids of the mouth might not directly injure the teeth, the state of the system which produces acid saliva affects the nutrition of the teeth, and thus impairs their power of resistance to external agents.

Dr. Ansart, who had lived in South America, stated that the natives of that country eat great quantities of acid fruit, and that their teeth soon become disintegrated and readily crumbled away.

Dr. Hawxhurst, a visitor from Michigan, was invited to take part in the discussion. He thinks that caries results from the combination of acids with the lime salts of the teeth, that the fluids of the mouth do not act directly on the teeth. We look for decay in fissures and depressions where food collects, and at the margin of the gum where acid mucous is secreted. The teeth of dyspeptics, whose secretions are so often acid, generally decay rapidly. The Dr. demonstrated by examples that temperature had but little influence on the teeth, the temperature of the mouth being uniform except when changed by the introduction of foreign substances.

Dr. Robbins said that the chemical theory of decay is the accepted one, the old theory being that decay proceeded from within outward; but now that it proceeds from without inward; thinks we have a combination of chemical and thermal agents causing decay by the decomposition of food lodged about the teeth. So the limestone of houses in eastern Pennsylvania crumble and disintegrate under the thermal and chemical influences of the atmosphere.

Teeth resist chemical action better than do the other animal tissues which are constantly changing; but the teeth have not the same power of repair which belongs to the other tissues. In order to understand the chemistry of the teeth, we must understand general chemistry. Lime salts should be administered through the food, as in brown bread, lime water in white bread, and directly, as hyphosphite of lime, etc.

Dr. Ansart mentioned that in South America a Chinese Coolie who would eat no fruit but lived on meat, bread and rice, had excellent teeth, while the other Coolies, who eat much acid fruit, had crumbling teeth, like those of the natives, yet the enamel in the first case was not so thick as that of the natives.

This closed the first days session. The discussion on Dental Chemistry was resumed on Wednesday, and Dr. G. Elliott being called to account, stated that he had omitted a part of his essay referring to gestation and lactation, on account of the presence of ladies in the audience.

Dr. Griffin thought this a false delicacy. It is the duty of the dentist to instruct the mother in these matters; thinks that acids injure the teeth, but that there must first be a door opened for them, which may be accomplished in the mother during gestation, when the child takes part of her nutriment

Dr. McDonneld said that deterioration of the teeth results from diseased constitutions, the effect of improper living; people must be taught to live simply and correctly, or remedies and diet will be of little avail; illustrated his position by an example from Dr. Burdell's book, viz.: The teeth of cows fed on their natural food are sound, while the teeth of those fed on hot swill are much diseased. True medicine is proper living, which in a few generations can regenerate the human race.

Dr. Wallace said that any of the acids formed in the mouth can decompose the teeth especially when there is not good resisting power. These acids produce chemical changes.

Nitric acid attacks the albumen of the teeth, and causes brown decay. Sulphuric acid produces black and hydrochloric acid white or chalky decay. These acids are generated by a disordered stomach, and other causes. When there are fillings of different metals in the same tooth, a battery is formed and a galvanic current flowing from the baser to the finer metal, producing decay. This should be guarded against. Not more than once in five hundred times should we use any other metal than gold for filling teeth.

Dr. Griffin asked if the enemy outside could force an entrance without the assistance of a traitor within.

Dr. Wallace replied that we may take a sound tooth and muriatic acid and produce decay out of the mouth; still, if a man's health was perfect, he would probably have no decayed teeth, the vital force enabling them to resist external agencies.

Dr. Allen had lived in the South Sea Islands; had never known a native to have the toothache, but where he was there were few acid fruits. The health of these islanders was generally good; they had no fevers, but had pulmonary diseases; they took great delight in bathing. Among the Indians of Michigan he found many bad teeth, but they lived much as the white men did.

Dr. Wallace had lived in Valparaiso, and found the teeth of the South Americans all more or less decayed. The half breeds were much afflicted. He came to the conclusion that the direct cause was a hot drink called Matte. The front teeth were most affected. Those who used improper food had a different decay; those articles which had sulphur in them formed sulphurous acid, which, taking oxygen from the atmosphere, formed sulphuric acid, and this produced decay.

Discussion closed.

MANAGEMENT OF CHILDREN'S TEETH.

Dr. G. W. Nelson read a paper on this subject, in which he advocated the use of prophylactics, the filling of deciduous teeth with gold, Hill's stopping, etc , and educating the parents in the care of their children's teeth.

Dr. Carroll stated that at its last meeting the American Dental Association had appointed a committee to draw up short rules for the management of children's teeth, and have them inserted in school text books.

Dr. Carroll said we never should extract the deciduous teeth before the time of the eruption of the permanent teeth if it is possible to avoid it. Would treat exposed nerves and fill as in permanent teeth. For filling these temporary teeth he likes tin, which can be put in quickly; after tin Hill's stopping, which must be renewed occasionally. Has extracted before the time for the eruption of the permanent teeth when there are well defined cases of alveolar abscess; thinks there is a tendency to contraction of the jaw on the premature extraction of deciduous teeth.

Dr. G. Elliott finds great difficulty in using gold or tin in deciduous teeth; thinks in some cases amalgam well prepared makes as good a filling for deciduous teeth as we can get; does not advocate the general use of amalgam; uses oxychloride of zinc most.

Dr. Ansart never uses amalgam in children's teeth; prefers Hill's stopping and Guillios' cement.

Dr. Wallace—One condition of deciduous teeth is where the permanent teeth appear before the deciduous teeth are properly disposed of. Permanent teeth come in a wrong position.

Dr. Robbins—Management of children's teeth includes the permanent teeth. Six year molar is usually the first tooth coming under the dentist's care. Parents often think it a temporary tooth and neglect it. We should treat and preserve this molar as well as all other permanent teeth; thinks that the shortening of the jaw and change of facial angle of the Anglo-Saxon race is caused by the early extraction of permanent teeth. Nature's model is the best; we can not restore the contour of the face after the extraction of cuspids; should avoid amalgam if possible; but would use it rather than extract.

Dr. C. D. Elliott has better success with cement plombe than any other plastic filling. Gave a case of nine months standing, which is still good.

Dr. McDonneld mentions an oxychloride of zinc filling, which has lasted two years. The density of *os-artificiel* depends on the method of manipulating it.

Dr. Ansart called for information concerning the lancing of children's gums.

Dr. Griffin—When the gums are tumefied, inflammation high, and children almost in convulsions, would not hesitate a moment to lance. Thinks there is much virtue in Homœopathic medicines in these cases.

Dr. Ansart would invariably lance when the membrane is tense and the inflammation is high, passing a sharp lance directly down to the tooth on the line of the cutting edge.

Dr. Dunn said that cicatrices yielded more readily to pressure than normal tissues, so that lancing did not retard the eruption of the tooth.

Dr. Elliott thought that more pain proceeded from the tension than from congestion.

Dr. Wallace said that the congestion was caused by pressure. The arrested blood corpuscles pressing on the nerve tissue cause pain. This condition was relieved by lancing.

### ALVEOLAR ABSCESS.

Dr. Carroll read an essay on Alveolar Abscess, describing its various forms and the treatment required. His rule for applying local remedies, was to choose that most agreeable to the patient, whether hot or cold. Discussion followed. To illustrate, Dr. Carroll took the case of a central incisor.

Would inject an escharotic. When all inflammation was subdued and the fistula healed, would fill the root solidly, and, if the trouble is renewed, would open through the alveolus, and remove the sac mechanically.

Dr. Templeton has had more success with this method than with any other, but fills before treating. Treatment through the canal is not effective unless we can force the creosote through the fistulous opening. After breaking up the sac, applies creosote. Sometimes uses an anæsthetic, before operating. If this treatment fails, it is because we do not reach the spot when opening through the gum.

Dr. McDonneld has been treating abscess for six years, and is confident that he has cured 99 in 100 cases in the superior maxilla. In the inferior maxilla is not so successful. Injects an escharotic, usually creosote, through the pulp canal. Almost all cases of failure are due to an impaired condition of the blood, or as in the inferior molars, to the difficulty of reaching the sac. The escharotic changes conditions and destroys life, the absorbents then carry off the effete matter if the person is healthy.

Dr. Hawxhurst wanted information about constitutional conditions. The same treatment will succeed in one case and in others fail. We can not cure abscess, but only change conditions while nature cures. In some constitutional conditions, nature can not do this.

Dr. Carroll—Medicine never cures anything. Nature, vital force, the "little workers" cure. If the workers are sick, they can not perform this work. We must first bring the little workers the blood corpuscles, in good condition.

Dr. Robbins remembers when it was considered unprofessional to treat alveolar abscess, and when he was called a fool, by professional men for attempting it. We must use the term cure for want of a better one. In some cases apparently healthy, it is difficult to decide why we do not cure. In others a constitutional taint, as syphilis is easily detected. When the foramen of the canal is small, and there is no fistula, must make an opening through the gum. The best instrument for this purpose is a sharp Gates' Drill, which goes through the process easily and brings out the cuttings, leaving a smooth, round opening. When after apparently cured, inflammation supervenes, may often arrest it by counter irritants on the gums. The action of iodide of potassium in these cases, is illustrated in Dr. Watt's Chemical Essays. The iodine and potassium are like coupled dogs harmless till they reach the point of obstruction, when they are released. The iodine goes off with oxygen as iodic acid, while the caustic potash dissolves the obstructions.

Dr. Geo. Elliott considers the treatment of alveolar abscess in the same light as other surgical operations. After breaking down the sac, introduce remedies which dissolve the material and prepare it to pass out, then keep the fistula open till the cavity is filled up by healthy granulations proceeding from the bottom to the top, assisting with tonics. Several interesting cases were described by Drs. Griffin and McDonneld. Discussion closed.

### MECHANICAL DENTISTRY.

A paper on Mechanical Dentistry, read by Dr. Herrick, next gave rise to some discussion.

Dr. Templeton held that to obviate air holes in plaster models, soak the impression half an hour in water, having thinly coated it with varnish to give color, and pours without oiling.

Dr. Pierce uses aniline in the water to color impressions, and uses silex for parting.

Dr. C. D. Elliott when taking impressions, uses the plaster thin if the palate is spongy, so as not to push away the soft parts. If the parts are firm mixes the plaster thicker. When taking impressions for partial sets, if the natural teeth are ill shaped, oils the impression cup before mixing the plaster. After taking the impression, removes the cup first and splits the plaster before removing it.

Dr. McDonneld thinks that the expansion of impressions results from having too thick a body of plaster, to be remedied by using wax first and over that a thin coating of plaster. After taking the impression puts it in water to prevent heating which causes expansion.

Dr. Dunn thinks expansion takes place in the vulcanizer.

Dr. Templeton—To get a good articulating bite, directs the patient to sit upright and stretch the muscles of the neck before closing the jaws.

Dr. Ansart—For partial sets takes impressions before extracting roots, and carves out the plaster—getting good results.

Dr. C. D. Elliott has put up one set of teeth on the Hyatt Perkins base. If it will withstand the fluids of the mouth, and wears well, it will supersede rubber. Put up the case in 1½ hours.

Dr. Wallace describes the method of using pyroxyline, which he prefers to rose pearl. It is composed principally of gun cotton and ether submitted to heavy pressure which forces out the ether. For soldering it, fillings of the material itself reduced to a pulp by ether, are used.

Dr. Robbins—The Hyatt Perkins base contains camphor. Is afraid of the effect of camphor on the mucous membrane. This base is inflammable as is the case with all collodion bases.

Dr. Tenny has cast aluminum plates The process requires great nicety of manipulation to obviate the effects of shrinkage when cooling. After casting, must jar the flask and plunge two-thirds of it into wet sand where it must be rotated continually, the plunger being pressed hard against the mouth of the flask. The upper part of which must be kept warmer than the lower. Aluminum melts at about 1,500°, gets better fitting plates of aluminum than of rubber.

Dr. Hawxhurst swages thin plates of aluminum on type metal dies, the shrinkage of which is about right. Fastens the teeth with Watt's & Williams metal, columbium. Drills holes in the alveolar ridge, countersinks, and fills them with pure wax. Proceeds as for a rubber case, using powdered pumice in the plaster to prevent shrinkage; heats the flask almost to the melting point of the metal; then having the metal as nearly as possible at the melting point, pours the metal and jars the flask.

Dr. Tenny suggested the use of parafine instead of wax to fill the countersinks, as it will disappear entirely when heated.

Dr. Robbins gave Prof. Barker s method of soldering aluminum plates. Prof. Barker swages the plate, scrapes it clean, and melts on it a little solder or tin, over a spirit lamp and usually gets a good union, but sometimes fails. Then heating the invested plate, he casts the metal in. In this method does not pierce the plate. Dr. Robbins thinks that the use of the French flux naphtha which is a hydrocarbon without oxygen, protects the plate from oxygen, and thus allows a union. Dr. R. stated that Dr. Moffitt, of Harrisburg, has improved his non-sectional block work. The sections are now molded and biscuited before the body is packed around them.

Dr. Hawxhurst advocated the use of gold as a base for artificial teeth, it being a good conductor of heat, incorruptible, and easily swaged, can be attached to the teeth by rubber. The objection to it is its high price. No material will ever be very successful which is not a good conductor of heat.

Dr. Templeton prefers Allen's Continuous Gum, Moffitt's Non-sectional Block Work, and the Looms patent, to gold.

Dr. Robbins thinks the safest and best way to use rubber is as an attachment to gold plate. Passes a continuous rim of gold around inside the teeth; does not perforate the plate but solders gold loops to the plate to hold the rubber. This makes the best denture, being clean and strong, and the thermal changes are good.

Dr. C. D. Elliott uses double headed platinum pins instead of gold loops. Is experimenting with plumpers, and if successful, will report at the next meeting.

Dr. Robbins said that the first plumpers he ever saw was made of cotton. Gave an interesting account of his early experience in working gold in old times, when he was obliged to melt his gold at the blacksmith's forge, for which privilege he was accustomed to blow the bellows for the blacksmith.

Dr. Harrison gave his method of resuscitating patients who are sinking under the use of chloroform. He passes the feather end of an uncut quill down the throat and gives it a rotary motion. The patient will revive instantly, there being sympathetic connection between the glottis and the heart. Has always succeeded in this way.

The session of Thursday commenced with discussion of an essay read by Dr. McDonneld on

### The Necessity of Dividing the Dental Profession into Two Distinct Specialties—Operative and Mechanical—and the Benefit to be Derived Therefrom.

Dr. G. Elliott thought that the division of the profession would make it the interest of the mechanical dentist to destroy the natural teeth.

Dr. McDonneld explained that he would take, by law, the forceps out of the hands of the mechanical dentist.

Dr. Tenney states that, in his town, the physicians extracts more teeth than the dentists.

Other members made the same statement.

Dr. G. Elliott thinks that for every tooth extracted by dentists, physicians extract 100. Advocates the passage of a law dividing the profession and compelling dentists to obtain a diploma before commencing practice.

Dr. Pierce shows his patients Dr. Bond's work on anæsthetics and their effects, and tells them that every time they take anæsthetics or have a tooth extracted, they have driven a nail in their coffins.

Dr. Robbins says we are now suffering from the inroads of physicians. There are short roads to the title of M. D., and incompetent men are continually slaughtering teeth.

When trying to get the passage of a law regulating the practice of dentistry, there was at first, a clause allowing physicians to extract teeth, but this was afterwards stricken out.

There was at first good prospects of the passage of the law, but it was soon clogged. Petitions asking for its passage were then circulated among the dentists of the State, and in this section, at least, were very generally signed. Another attempt to secure the passage of the law will be made at the next session of the Legislature.

The following is a copy of the proposed law:

AN ACT to improve and regulate the practice of Dentistry in the State of Pennsylvania.

Sec. 1. Be it enacted by the Senate and House of Representatives of the State of Pennsylvania in General Assembly met, that it shall be unlawful for any person to practice dentistry in the State of Pa., for compensation, unless such person has received a diploma from a reputable dental college, duly incorporated under the laws of this or some other State of the United States, or foreign country, or has obtained a certificate of qualification issued by the State Dental Society, as hereinafter provided, or has been in reputable dental practice for ten or more consecutive years in this State: Provided that nothing in this section shall apply to persons now engaged in the practice of dentistry in this State before the 1st day of January, A D. 1874.

Sec. 2. It shall be the duty of the State Dental Society at its first annual meeting after the passage of this act, and annually thereafter, to elect five qualified members of the State Dental Society to act as a board of examiners, whose duty it shall be to meet at least once a year to examine all applicants, and, upon a satisfactory examination, issue a certificate of qua'ification t) practice dental surgery.

Sec. 3 To provide a fund to carry out the provisions of Sec. 2, the board of examiners shall collect from all who receive certificates to practice Dental Surgery, the sum authorized by the State Dental Society, not exceeding Thirty dollars each, and account to said society for the same.

SEC. 4. Any person who shall practice dentistry without having complied with the requirements of this Act, shall be deemed guilty of a misdemeanor, and upon conviction thereof shall be fined not less than fifty (50), nor more than two hundred (200) dollars.

SEC. 5. All prosecutions under this Act shall be by indictment before the Court having jurisdiction in the county where the offence was committed, and all fines imposed and collected under the provisions of this Act, shall be paid into the treasury of the school fund in the district where the offence was committed, for library purposes.

Dr G. W. Nelson: In Ohio there is a State law regulating the practice of dentistry, and it is working well.

Dr. Wallace, who had been on the committee to obtain legislative action in Ohio, related his experience, and thought that it was an advantage to have the matter delayed, that it may be agitated and discussed, and the minds of the people aroused.

On motion of Dr. Templeton a rising vote was taken for and against the passage of a law regulating the practice of dentistry, resulting unanimously in favor of the passage of such a law.

### SALIVARY CALCULUS AND ITS TREATMENT.

A paper on this subject, read by Dr. J. H. Nelson, gave rise to the following discussion:

Dr. Nelson formerly cleaned teeth for two shillings, but now can obtain from two to four dollars, so great is the benefit derived from the operation.

Dr. Pierce advocated extreme care in removing every particle of tartar from beneath the gums, showed how to use the tooth brush with a vertical motion, afterwards passing waxed floss silk beneath the gums and around the teeth.

Dr. C. D Elliott, after removing tartar, always polishes the teeth with powdered pumice, and burnishes them.

Dr. Templeton thinks that the essayist's treatment of the gums, after cleaning the teeth, viz , the application of a solution of chloride of zinc, 40 grains to the ounce of water, is too powerful. Prefers creosote.

Dr. G. Elliott has seen Dr Nelson apply the chloride of zinc with excellent and rapid effect.

Dr. Robbins says perfectly cleaned teeth will not decay, but imperfectly cleaned teeth are in a worse condition than those not cleaned at all. Discourages the use of powders. Does not use tincture of myrrh, preferring tannin, and has used chloride of zinc, but is very cautious, as the hydrochloric acid will leave the zinc and attack the tooth. For diseases of the gum and suppurating peridental membrane, however, chloride of zinc is an excellent remedy after the failure of creosote. Finds a difference in the action between carbolic acid and pure beachwood creosote, the latter acting more kindly on the pulp and peridental membrane. Discussi n closed.

OPERATIVE DENTISTRY.

Dr. Bagley read some notes on operative dentistry, calling attention to a few points in practice—as the use of Guillois' cement, Pack's Eureka gold, light and heavy foils, material for mallet, etc Discussion followed.

Dr. Hawxhurst likes Guillois' cement—should apply creosote to the cavity, dry it, and then apply the cement, at first thin, afterwards mixed thick and applied quickly. Keep dry till hard; coat with very thin sandarac varnish to fill the pores, and then polish. Dr. C. D. Elliott protects the pulp from the caustic action of Guillois' cement with plaster of paris and thin carbolic acid.

Dr. Carrol uses Palmer's continuous current electric battery, on inflamed surfaces, and in chronic alveolar abscess. Is very successful with the battery when treating sensitive dentine. If the battery were powerful enough, could overcome every case. Applies one pole over the tooth, and the other over the nerve ganglia. Has feared inflammation of the pulp, but has not aroused it with the electricity.

Dr. Robbins has used electricity since 1858. This use of electricity was patented—one pole being attached to the forceps, and the other in the hand of the patient. Tried this but did not like it. Made a saddle to embrace the gum, and found it invaluable when cutting sensitive dentine. Now uses a brass button attached to the pole. This is placed on the gum over the tooth. The other pole is usually placed in the patient's hand. This is very useful when extracting.

When electricity is applied directly to the tooth, it is more unpleasant than when applied to the gum. Has more direct and concentrated current when the pole is applied to the ganglia than when it is held in the hand.

When applying the rubber dam, it is often convenient to use the string in a half hitch. Another way to hold rubber in place, is by a wedge pressed between the teeth by a perforated forceps. The rubber dam is the best protection to the dentist against fœtid breath of patients. Dr. Butler, of Cleveland, always applies the rubber dam before beginning to excavate; can remove the debris with a dry syringe. To avoid danger of periostitis and abscess from the forcing of fluids through the foramen at the apex of the root, when filling, use the hot air syringe, which will dry out the root canal better than anything else.

For trimming down approximal fillings finds a sickle shaped, knife-edged instrument very useful. Rough cloth gives a good finish. Chalk gives the best final polish.

After filling on the grinding surface, always cuts down the gold until the teeth can close without striking the gold, before allowing the patient to leave the office.

Saliva, when exposed to the air, oxidizes so rapidly that there is great danger of making mistakes when testing.

Advocates the use of steel as a material for mallets. Likes nickel plating to prevent rusting. Uses thin shellac varnish for the same purpose.

Dr. Hawxhurst suggested the use of weak collodion to prevent rusting of instruments; the weaker the better, provided a film is formed.

Dr. Ansart says that Dr. Harvey, of Buffalo, when applying the rubber dam over lower molars, in difficult cases, attaches a small piece of leather to the string, and places it so that the leather rests against both the rubber and the tooth, and prevents the string and rubber from slipping.

Dr. Ansart described Bonwell's electric mallet also, an instrument which can be attached to any plugger, and by means of which gold can be used in long strips.

Dr. Hawxhurst said the principal use of Green's pneumatic engine was for trimming down gold and cutting retaining pits—the pneumatic plugger was not so successful.

Dr. McDonneld, after filling approximal cavities, tries to leave no space between the teeth after the operation is finished; otherwise food will lodge between the teeth and be very troublesome.

Dr. Hawxhurst spoke of teeth worn down by occlusion, and asked for a remedy.

Dr. McDonneld always fills such teeth with gold.

Dr. Wallace had such a case where the superior incisors were worn away, and the inferior incisors beveled very thin and sharp. He built up the bicuspids, and cut down the inferior incisors 1-16th of an inch.

Dr. McDonneld says examination of teeth requires great care and nicety. Wedging will often show a cavity which would otherwise escape observation. Must use very fine instruments.

Dr. Robbins recommends making a list of cavities when the teeth are first examined and checking off as we fill.

Dr. G. W. Nelson related the case of a lady who had her teeth examined, just before leaving Europe, by a dentist who reported nothing to be done. Came home and had her teeth examined immediately, and soon after had fifteen cavities filled, some of them large.

Discussion closed.

ANNUAL ELECTION OF OFFICERS.

The following officers were duly elected for the ensuing year:

*President*—Dr. J. G. Templeton, of New Castle.

*Vice-President*—W. T. Wallace, of Kingsville, O.

*Secretary*—C. B. Ansart, of Oil City; *Treasurer*—J. H. Nelson, of North East.

*Censors*—A. B. Robbins, of Meadville; J. C. Gifford, of Westfield, N. Y.; W. T. Wallace, of Kingsville, O.

*Executive Committee*—G. B. McDonneld, of Conneautville; C. H. Bagley, of Meadville; F. Herrick, Greenville; J. H. Nelson, of North East; C. D. Elliott, of Franklin.

*Delegates to the State Dental Society*—F. Herrick, of Greenville; W. M. Martin, of Mercer; A. B. Robbins, of Meadville; J. G. Templeton, of New Castle; C. D. Elliott, of Franklin; J. B. Humes, of Edinboro; C. B. Ansart, of Oil City; E. M Pierce, of Warren, O.

*Delegates to the American Association*—Dr. C. H. Griffin, of Oil City; C. B. Ansart, Oil City; W. M. Martin, of Mercer; E. M. Pierce, of Warren, O.; D. C. Dunn, of Meadville; J. H. Nelson, of North East; J. C. Gifford, of Westfield, N. Y.; G. W. Nelson, of Ashtabula, O.

The executive committee decided on the following essayists and subjects for next year:

*Public Addresses*—Dental Education, Dr. A. B. Robbins; Mechanical Dentistry, S. W. Tenny; Operative Dentistry, especially Filling of the Teeth, C. D. Elliott; Treatment of Children's Teeth, W. M. Martin; Treatment of Diseased Gums, C. H. Griffin; Dental Pathology, W. T. Wallace; Treatment of Dental Pulps, D. C. Dunn; Why should we Extract Teeth? J. B. Humes; Dental Caries, W. E. Magill.

Censors reported that they had been unable to finish their labors, and asked to be continued, to report next year. Granted. Dr. G. Elliott moved that the next session be continued for three days, beginning on the first Tuesday in

May, and that the fact be mentioned in the Secretary's Circular. Carried.

On motion of Dr. G. Elliott, Dr. Hawxhurst was invited to deliver an essay before the association. Dr. Hawxhurst then read his essay on "The effects of Fœtid Breath on the general Constitution." A vote of thanks was tendered to Dr. Hawxhurst for his essay.

The Association then adjourned to meet on the first Tuesday in May, 1872, in Conneautville, Pa.

